# Biomechanical advantages of double-trajectory screws technique combined with multiple-rod constructs in lumbar pedicle subtraction osteotomy: a finite element analysis study

**DOI:** 10.3389/fbioe.2026.1827940

**Published:** 2026-08-03

**Authors:** Hu Lyu, Qiyang Wang, Hongbang Liu, Fengyi Jiang, Tiannan Zou, Qiunan Lyu

**Affiliations:** 1 Department of Orthopedics, The First People’s Hospital of Yunnan Province, The Affiliated Hospital of Kunming University of Science and Technology, Kunming, China; 2 The Clinical Medical Center for Spinal and Spinal Cord Diseases of Yunnan Province, Kunming, China; 3 The Key Laboratory of Digital Orthopedics of Yunnan Province, Kunming, China

**Keywords:** double-trajectory screws, finite element analysis, multi-rod constructs, pediclesubtraction osteotomy, rod fracture, screw pull-out

## Abstract

**Introduction:**

Although multi-rods constructs (Multi-RCs) have been demonstrated to reduce the risk of rod fracture (RF) following pedicle subtraction osteotomy (PSO), they offer limited improvement in terms of screw-related complications. The double-trajectory technique (combining traditional trajectory (TT) and cortical bone trajectory (CBT) screws) has been demonstrated to enhance screw fixation. Consequently, the employment of the double-trajectory technique in conjunction with Multi-RCs has the capacity to augment the fixation strength of both rods and screws. Thus, the study aimed to evaluate the biomechanical performance of a novel configuration combining double-trajectory screws with multi-rod in lumbar PSO.

**Methods:**

A validated L1-5 nonlinear FE model was modified to simulate a 25° PSO at L3. Three configurations were analyzed under both normal and osteoporotic conditions: (a) 2R-TT (one rod with TT screws on each side); (b) 4R-TT (one rod with TT screws on each side, supplemented by a lateral satellite rod); and (c) 4R-TT/CBT (one rod with TT screws and one rod with CBT screws on each side). Loads of 400 N and 7.5 Nm in various directions were applied, with L5 fixed. Outcomes measured included range of motion (ROM) and maximum von Mises stress on rods and screws.

**Results:**

The 4R-TT/CBT configuration had the smallest ROM in all directions, with reductions of 17.3%–26.4% compared to 2R-TT and 10.9%–16.8% compared to 4R-TT. It also showed the lowest von Mises stress on rods, particularly in flexion, where reductions ranged from 20.5% to 21.4% compared to 2R-TT and 20.9%–21.4% compared to 4R-TT. Considering the maximum von Mises stress on the screws, it exhibited the lowest and demonstrated a substantial reduction of 8.9%–22.8% and 19.5%–29.8% compared to the 2R-TT and 4R-TT configurations, respectively.

**Conclusion:**

The 4R-TT/CBT configuration provided superior biomechanical performance in lumbar PSO, enhancing construct stiffness, and substantially reducing stresses on rods and screws compared to the 2R-TT and 4R-TT configurations. However, this was a FE study with inherent simplifications. Therefore, the findings should be further confirmed by cadaveric or clinical studies in the future.

## Introduction

Pedicle subtraction osteotomy (PSO) is a prevalent technique that can achieve a sagittal correction of up to 25°–35° through a posterior approach without lengthening the anterior column. This procedure involves resection of the posterior and middle columns, while the anterior column acts as a hinge, allowing for correction without lengthening the anterior column (Ghobrial et al., 2017). It has been demonstrated to improve bone healing potential while reducing blood loss caused by the stretch of major vessels and viscera anterior to the spine. The technique has been employed to treat patients with fixed sagittal imbalance, including those with iatrogenic spinal deformity, adult spinal deformity, and ankylosing spondylitis kyphosis (Huang et al., 2020; Lee et al., 2020).

Although PSO can lead to significant improvements in radiographic and clinical outcomes, the incidence of rod fractures (RF) remains relatively high (9%–36.4%) due to increased rod strain after PSO (Zhao et al., 2021; Gehrchen et al., 2016; Lyu et al., 2022). RF following deformity correction can cause pain, neurological impairment, and loss of correction, often resulting in the need for revision surgery (Lee et al., 2020). To reduce the rate of RF, different types of multiple-rod configurations (Multi-RCs) have been developed (Lyu et al., 2022; Hyun et al., 2014; Gupta et al., 2018). They contain three or more rods that supplement the primary dual-rod fixation across mechanically demanding regions, such as PSO sites. The most common supplemental rods are satellite rods and accessory rods. Satellite rods are additional rods connected to the primary rods via side connectors, providing local reinforcement at areas of high rod strain. And accessory rods are short, independent rods anchored via their own pedicle screws above and below the osteotomy site, without direct connection to the primary rods. Several clinical studies demonstrated that Multi-RCs can significantly reduce the incidence of RF in comparison to a traditional two-rod configuration (Lyu et al., 2022; Hyun et al., 2014; Gupta et al., 2018). Nevertheless, while Multi-RCs can mitigate the strain on the rods, they are unable to do so for the screws. Consequently, screw pull-out and breakage may arise in patients who undergo PSO, particularly in those with osteoporosis (Kankam et al., 2024; Cho et al., 2022).

To enhance the fixation strength of screws, the cortical bone trajectory (CBT) screw technique has been proposed. In comparison to traditional trajectory (TT) pedicle screws, CBT screws demonstrate superior insertion torque, pullout strength, and fatigue strength (Santoni et al., 2009). TT screws and CBT screws have different entry points and directions. To combine their advantages, some studies have proposed placing both a TT screw and a CBT screw in the same pedicle. This is known as the double-trajectory technique (Mullin et al., 2016; Zhao et al., 2021). This technique has been demonstrated to enhance screw fixation strength, consequently reducing complications associated with screws and improving fusion rates (Ueno et al., 2013; Matsukawa et al., 2015).

Theoretically, the double-trajectory technique in conjunction with Multi-RCs has the potential to enhance the fixation strength of both rods and screws, thereby reducing the incidence of mechanical complications. However, there is currently a lack of biomechanical evaluations of the configuration in PSO models. Consequently, this study aims to perform a finite element (FE) analysis to compare the mechanical properties of the configuration comprising double-trajectory screws and multiple rods to other conventional fixation configurations for the PSO procedure under normal and osteoporotic conditions.

## Materials and methods

### Establishment of the standard finite element lumbar spine model

A three-dimensional nonlinear FE model of the lumbar spine from L1 to L5 was constructed from the CT scan of a 60-year-old female volunteer with no spinal disease. The original data in DICOM format were collected and imported into Mimics 21.0 (Materialise, Belgium, RRID: SCR_012153) to reconstruct the corresponding 3D geometric model. Subsequently, the model was exported to a data file in STL format and imported into Geomagic 2021 (Geomagic, United States, RRID:SCR_016978) for surface optimization and surface fitting processing. Thereafter, the model was imported into SolidWorks 2021 (Dassault Systems, United States, RRID:SCR_024908) for assembly and defect processing. Finally, it was imported into Ansys 2021R1 (Ansys, United States, RRID:SCR_022135) for meshing and material assignment. The methodology employed for modeling is consistent with that utilized in the previous studies (Lu et al., 2022; Zhang et al., 2022). The established FE model of the lumbar spine from L1 to L5 was utilized to simulate the surgical scene.

### Material properties and validity verification

**FIGURE 1:**
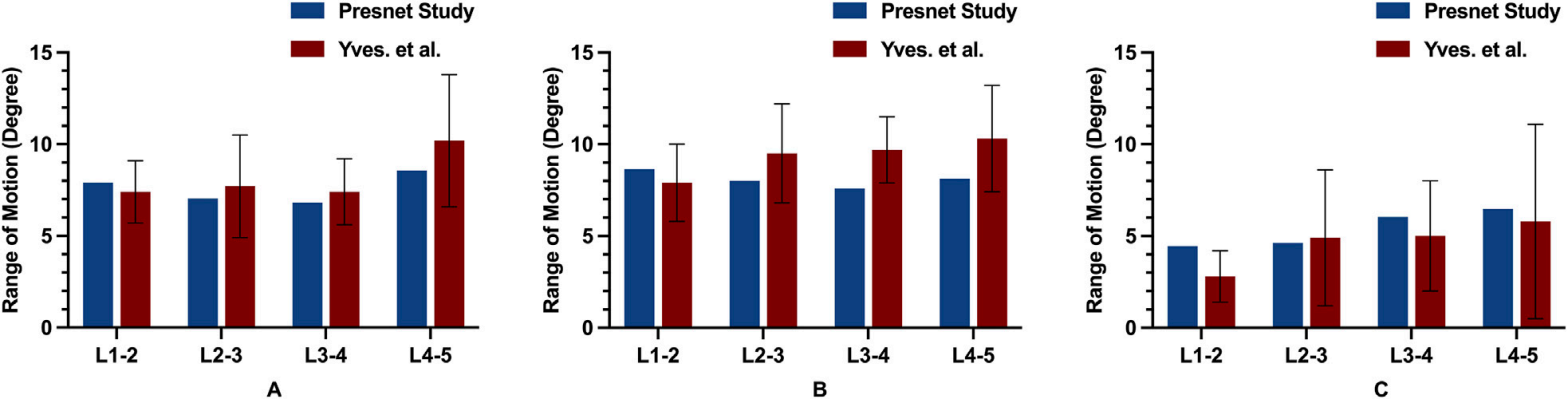
Comparison of the segmental ROMs of the established model with those in the previous cadaveric study. **(A)** Flexion and Extension. **(B)** Lateral Bending. **(C)** Axial Rotation.

The material parameters were derived from the literature (Table 1) (Lu et al., 2022; Zhang et al., 2022; Liu et al., 2024). In the study, the thickness of cortical bone was 1 mm, and the thickness of the endplate was 0.5 mm (Lu et al., 2022; Liu et al., 2024; Moran et al., 1989). The upper and lower endplates were connected seamlessly with the surfaces of the vertebral body. The intervertebral disc was composed of a nucleus pulposus and an annulus fibrosus. The nucleus pulposus constituted 40% of the total volume of the intervertebral disc (Lu et al., 2022; Sun et al., 2023). The linear elasticity model was applied to the bone and cartilage structures. The reconstruction of the spinal ligaments was simulated using springs elements that resist tension only. The Young’s modulus of the screws and rods was set at 110,000 MPa. The osteoporosis model was established with reference to the published literature (Liu et al., 2024; Bereczki et al., 2024; Polikeit et al., 2003). The Young’s modulus and density of the osteoporotic cancellous bone were reduced by 67%, and those of cortical bone were decreased by 34% (Liu et al., 2024; Guo et al., 2025). Validation of the finite element model was performed by comparing the predicted segmental ROM with cadaveric data from Ntilikina et al. (Ntilikina et al., 2020). In the validation, the intact L1–5 finite element model was subjected to a compressive preload of 400 N applied to the superior endplate of L1 (maintained in a fixed vertical direction), followed by pure moments of 7.5 Nm applied in four directions: flexion, extension, left lateral bending, and left axial rotation. The L5 inferior endplate was fully constrained in all degrees of freedom. The percentage differences between the FE-predicted and cadaveric mean ROM values ranged from 5.7% to 16.0% across all levels and loading directions, with a mean difference of 10.7% (Figure 1).

**TABLE 1:** Material properties of the finite element model.

Component	Young’s modulus (MPa)	Poisson ratio	Cross-sectional area (mm^2^)
Cortical bone (normal)	12,000	0.3	—
Cortical bone (osteoporotic)	8040	0.3	—
Cancellous bone (normal)	100	0.3	—
Cancellous bone (osteoporotic)	34	0.3	—
Endplate	24	0.4	—
Annulus fibrosus	4.2	0.45	—
Nuclear pulposus	1	0.49	—
Cartilage of facet joints	10	0.4	—
Screws and rods	110,000	0.3	—
Anterior longitudinal ligament	7.8	0.3	22.4
Posterior longitudinal ligament	10	0.3	7.0
Interspinous ligament	10	0.3	14.1
Supraspinous ligament	8	0.3	10.5
Intertransverse ligament	10	0.3	0.6
Ligamentum flavum	17	0.3	14.1
Capsular ligament	7.5	0.3	10.5

### Establishment of the lumbar PSO models with different fixation configurations

**FIGURE 2:**
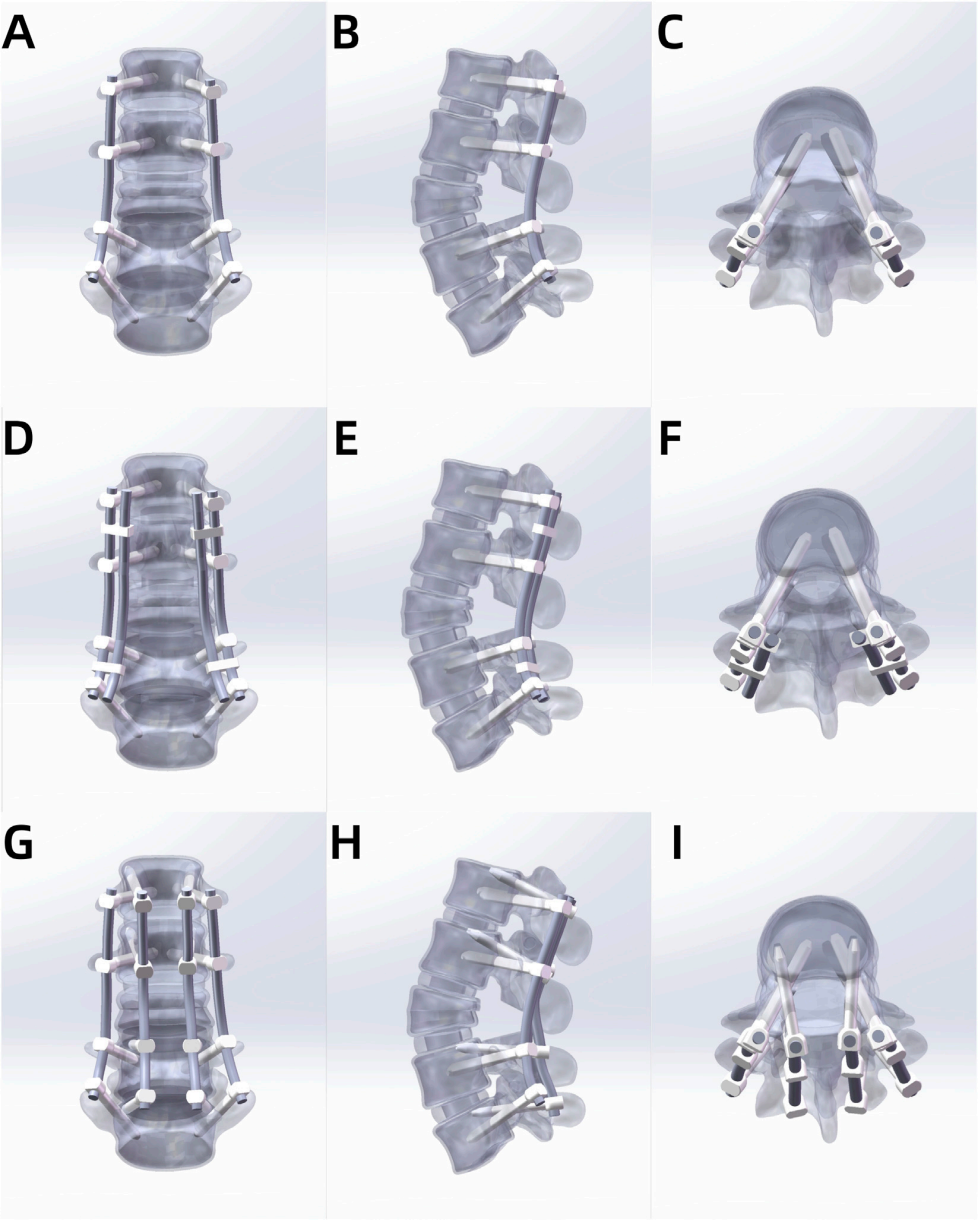
Posterior, lateral and axial view of the three fixation configurations applied to the lumbar PSO model. **(A–C)** 2R-TT (one main rod connected to TT screws on each side). **(D–F)** 4R-TT (one main rod connected to TT screws on each side, supplemented by a satellite rod attached laterally by cross-links). **(G–I)** 4R-TT/CBT (one rod connected to TT screws and one rod connected to CBT screws on each side).

Consistent with the osteotomy technique used in previous studies, the FE model of the lumbar spine was modified with a 25° PSO at L3 (Berjano et al., 2019; Januszewski et al., 2017). The osteotomy was created by removing all the posterior elements of L3. Following the resection of pedicles and facet joints, a wedge-shaped osteotomy of 25° was performed at the vertebral body. The lordosis of L3 was −2° pre-osteotomy and 23° post-osteotomy. The lordosis of L1-5 was 24° pre-osteotomy and 49° post-osteotomy. Three distinct fixation configurations were then applied to the lumbar PSO model: (a) 2R-TT (one main rod connected to TT screws on each side); (b) 4R-TT (one main rod connected to TT screws on each side, supplemented by a satellite rod attached laterally by cross-links); and (c) 4R-TT/CBT (one rod connected to TT screws and one rod connected to CBT screws on each side) (Figure 2). The TT screws had a diameter of 6.0 mm, and a length of 45 mm. The CBT screws had a diameter of 5.5 mm, and a length of 40 mm. The rods had a diameter of 5.5 mm. The entry point of the CBT screw was defined at the intersection of the midline of the superior articular process and the horizontal line situated 1 mm below the inferior margin of the transverse eminence process. The trajectory of the CBT screw was 25°–30° toward the head and 10° toward the outside (Chang et al., 2021). In all configurations, the rods were contoured to conform to the three-dimensional spatial positions of the screw heads to which they were connected. The main rods (connected to TT screws) were generated using identical screw head positions across the 2R-TT, 4R-TT, and 4R-TT/CBT configurations, ensuring that the main rod geometry and contour were identical in all three constructs. In the 4R-TT/CBT configuration, the medial rods were independently contoured to match the distinct spatial positions of the CBT screw heads, which differ from the TT screws in terms of entry point and trajectory. No additional pre-bending or pre-strain was applied to any of the rods, the contour was purely defined by the screw head positions. Titanium alloy (Ti-6Al-4V) was selected for the screws and rods. The interconnections between the rod and crosslink, the screw and vertebra, and the screw and rod were modeled as bonded contacts, which prohibit any relative movement between the contacting surfaces. The contact between the two resected extremities was also modeled as a bonded contact.

### Loading and boundary conditions

The same loading and boundary conditions were applied to the three fixation configurations in both the normal and osteoporotic models. First, a compressive preload of 400 N was applied to the superior endplate of the L1 vertebra to simulate the gravitational load of the upper body. This force was maintained in a fixed vertical direction aligned with the global vertical axis regardless of spinal motion. Then, pure moments of 7.5 Nm were applied to the upper endplate of L1 in four anatomical directions, including flexion, extension, left lateral bending, and left axial rotation. During the simulation test, the inferior endplate of the L5 vertebra was constrained in all degrees of freedom. The global ROM of L1-5, the maximum von Mises stresses on the rods and screws were measured and compared for all configurations.

## Result

### Range of motion

**FIGURE 3:**
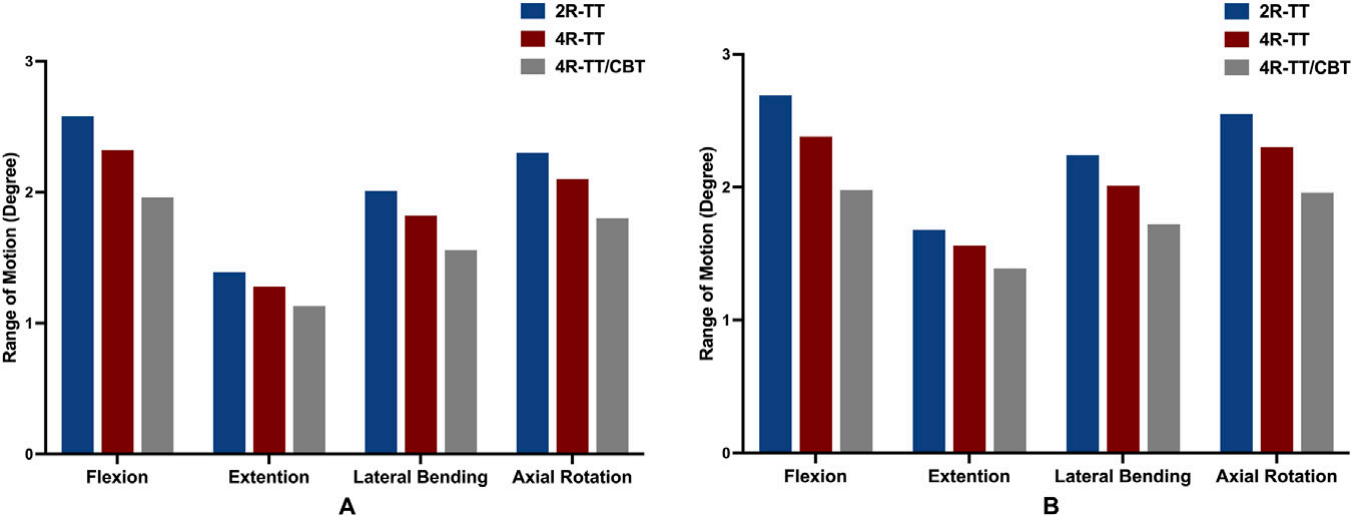
Comparison of the global ROM of L1-5 for different loading directions and configurations under both normal **(A)** and osteoporotic **(B)** conditions.

In the normal models, the ROMs of the 4R-TT/CBT configuration were the smallest in flexion, extension, left lateral bending, and left axial rotation motions among all three groups. Compared to the ROMs of the 2R-TT configuration (2.58°) and the 4R-TT configuration (2.32°) in flexion, the 4R-TT/CBT configuration (1.96°) showed a reduction of approximately 24.0% and 15.5%, respectively. In comparison to the ROMs of the 2R-TT configuration (1.39°) and the 4R-TT configuration (1.28°) in extension, the 4R-TT/CBT configuration (1.13°) exhibited a reduction of approximately 18.7% and 11.7%, respectively. Compared to the ROMs of the 2R-TT configuration (2.01°) and the 4R-TT configuration (1.82°) in left lateral bending, the 4R-TT/CBT configuration (1.56°) showed a reduction of approximately 22.4% and 14.3%, respectively. A comparison of the ROM of the 4R-TT/CBT configuration (1.80°) in left axial rotation with those of the 2R-TT configuration (2.30°) and the 4R-TT configuration (2.10°) revealed a reduction of approximately 21.7% and 14.3%, respectively (Figure 3).

Compared to normal models, the ROMs increased by 1%–23.0% in all loading directions for all three groups (0.02°–0.29°) under osteoporotic conditions. The most substantial increase, by 20.9%–23.0%, was observed in extension (0.26°–0.29°). The discrepancies in the ROMs among all three configurations were also further amplified in the osteoporotic models, with a range of 9.1%–25.0% (Figure 3).

### von Mises stress on the rods

**FIGURE 4:**
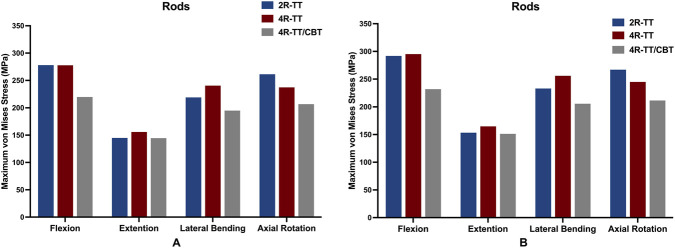
Comparison of maximum von mises stress on the rods for different loading directions and configurations under both normal **(A)** and osteoporotic **(B)** conditions.

**FIGURE 5:**
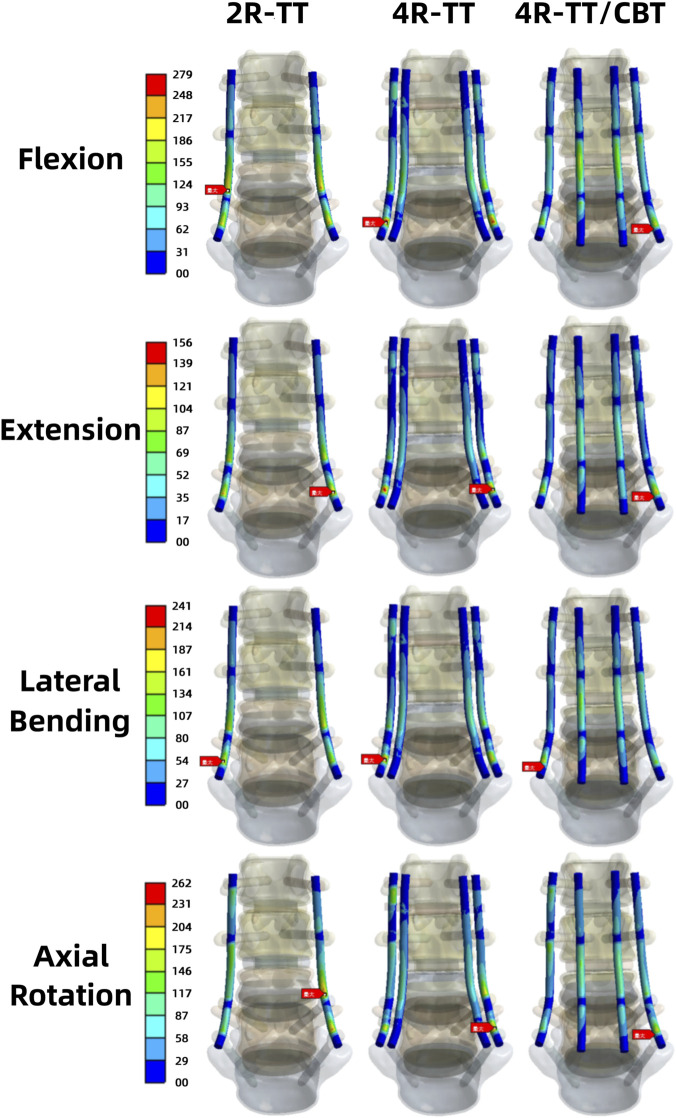
Comparison of von Mises stress distribution on the rods for different loading directions and configurations under normal condition. The color bar represents stress values in MPa. The maximum stress location is marked in red.

In the normal models, the maximum von Mises stress on the rods occurred with flexion in all the configurations. Compared to the 2R-TT and 4R-TT configurations, the 4R-TT/CBT configuration exhibited the lowest von Mises stress on the rods and demonstrated a substantial reduction (0.2%–21.0%) in all the four loading directions. In flexion and left axial rotation, the 4R-TT configuration had lower rod stresses than the 2R-TT configuration. However, in extension and left bending, the 4R-TT stresses were higher than those of the 2R-TT configuration (Figure 4). The maximum von Mises stress on the rods in the 4R-TT and 4R-TT/CBT configurations were close to the L5 pedicle screws in all the loading directions. In the 2R-TT configuration, the maximum von Mises stress on the rods in extension and left bending were also adjacent to the L5 pedicle screws, whereas the maximum von Mises stress on the rods in flexion and left axial rotation were transferred to the location adjacent to the PSO level where the rods were highly contoured (Figure 5).

In the osteoporotic models, the maximum von Mises stress on the rods demonstrated an increase (2.2%–6.4%) in all the configurations compared to the normal models (Figure 4). However, the location of the maximum von Mises stress on the rods in the normal and osteoporotic models exhibited a comparable pattern.

### von Mises stress on the screws

**FIGURE 6:**
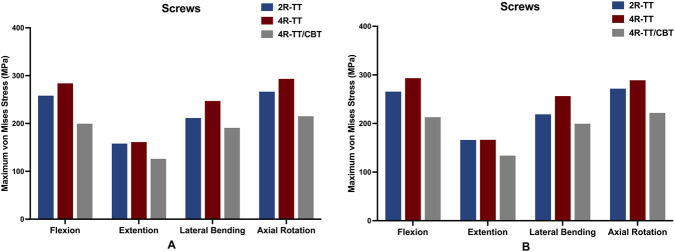
Comparison of maximum von mises stress on the screws for different loading directions and configurations under both normal **(A)** and osteoporotic **(B)** conditions.

**FIGURE 7:**
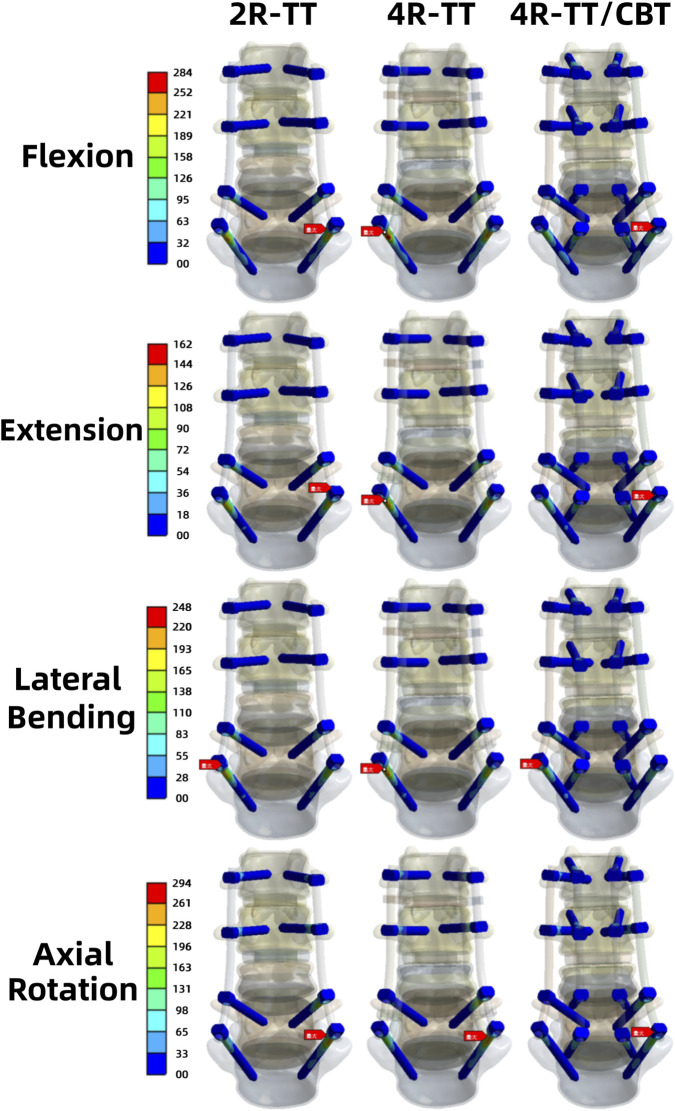
Comparison of von Mises stress distribution on the screws for different loading directions and configurations under normal condition. The color bar represents stress values in MPa. The maximum stress location marked in red. Magnified views of the L3–L5 region, where the most clinically relevant stress concentrations occur, are provided in the supplementary figure for improved clarity.

In the normal models, the maximum von Mises stress on the screws were observed with left axial rotation in all the configurations. The 4R-TT configuration exhibited the highest von Mises stress on the screws in all the four loading directions. And the 4R-TT/CBT configuration exhibited the lowest von Mises stress on the screws and demonstrated a substantial reduction (9.7%–29.8%) compared to the 2R-TT and 4R-TT configuration in all the four loading directions (Figure 6). In all the three configurations, the maximum von Mises stress on the screws were observed on the L5 pedicle screws which were the lowest screws in all the four loading directions (Figure 7).

In the osteoporotic models, the maximum von Mises stress on the screws demonstrated an increase (1.9%–6.8%) in all the configurations compared to the normal models (Figure 6). However, the locations of the maximum von Mises stress on the screws in the normal and osteoporotic models exhibited a similar pattern.

## Discussion

PSO is a common technique that has been utilized to treat patients with fixed sagittal imbalance (Ghobrial et al., 2017). However, the occurrence of mechanical complications (e.g., RF, screw loosening, screw pull-out, and screw breakage) associated with PSO procedures remains relatively high (Zhao et al., 2019; Lyu et al., 2022; Kankam et al., 2024; Cho et al., 2022). It has been demonstrated that Multi-RCs can reduce the rate of RF in comparison to a standard two-rod configuration (Lyu et al., 2022; Hyun et al., 2014; Gupta et al., 2018). However, they are unable to mitigate the strain on the screws and avoid relevant complications. The double-trajectory technique, which incorporates both the CBT and TT pedicle screws, has been demonstrated to enhance screw fixation strength (Mullin et al., 2016; Zhao et al., 2021; Ueno et al., 2013; Matsukawa et al., 2015). Accordingly, the double-trajectory technique in conjunction with Multi-RCs has the potential to enhance the fixation strength of both rods and screws, thereby reducing the incidence of mechanical complications. However, there is currently a dearth of biomechanical evaluations of the configuration in PSO models. Consequently, the present study compared the configuration comprising double-trajectory screws and multiple rods to other conventional fixation configurations for the PSO procedure under normal and osteoporotic conditions.

The ROM of the FE lumbar spine model can be utilized to reflect the stiffness of the fixation configuration. The efficacy of satellite rods in reducing motion at the PSO level has been demonstrated by both *in vitro* and finite element (FE) studies (Berjano et al., 2019; Hallager et al., 2016; Seyed Vosoughi et al., 2019). The results of the current study were consistent with these findings. In the present study, the ROMs of the 4R-TT configuration showed a reduction of 7.9%–10.1% compared to the 2R-TT configuration in the four motions (Figure 3). Although our study and those studies share the same basic satellite-rod topology (satellite rods connected to the main rods via side-by-side connectors), we acknowledge that direct cross-study comparisons of ROM are limited by differences in rod material, anatomical range, and loading conditions. Nevertheless, all studies consistently demonstrate that satellite rods substantially reduce ROM compared to 2R-TT configuration. Matsukawa K et al. demonstrated that the double-trajectory technique can achieve rigid fixation strength by combining two different trajectories to obtain triangulation effects in both the sagittal and horizontal planes (Matsukawa et al., 2015). Our results were in accordance with this finding. In the present study, the ROMs of the 4R-TT/CBT configuration were the smallest in the four motions among all the three groups. Furthermore, the discrepancies between the ROMs of the three configurations were further amplified by osteoporosis (Figure 3). The results indicated that the 4R-TT/CBT configuration exhibited the most rigid fixation strength compared to the other two configuration. And it is likely to gain favorable outcomes in the treatment of PSO cases with osteoporosis.

In terms of von Mises stress on the rods, the results of the current study demonstrated that the 4R-TT configuration (satellite-rod configuration) resulted in a reduction of primary rod stress at the PSO level in comparison to the 2R-TT configuration. This can be attributed to the implementation of multi-rods, which resulted in a substantial alteration to the moment of inertia of the implant, consequently leading to a reduction in stress on the single rod (Luca et al., 2017). However, the maximum von Mises stress on the rods of the 4R-TT configuration was found to exceed that of the 2R-TT configuration in extension by 7.5% (155.78 MPa vs. 144.87 MPa) and in left bending by 9.7% (240.45 MPa vs. 219.09 MPa). This finding was consistent with the conclusions of earlier studies. Berjano et al. found that satellite rods increased the maximum von Mises stress on the rods by 4% in extension, compared to the 2R-TT configuration (Berjano et al., 2019). Similarly, Ardalan et al. found that satellite rods increased the maximum von Mises stress on rods by 5% in extension and 11% in left rotation (Seyed Vosoughi et al., 2019). This can be attributable to the elevated global stiffness of the multi-rods configuration, in conjunction with the utilization of rod connectors which results in stress concentration on the primary rods near the connectors. As mentioned in the previous paragraph, although our study and those studies share the same basic satellite-rod topology, we acknowledge that direct cross-study comparisons of ROM are limited by differences in several factors. Nevertheless, all studies consistently demonstrate that satellite rods can increase maximum von Mises stress on rods compared to 2R-TT configuration in some directions. In contrast, the 4R-TT/CBT configuration featured direct connection of the satellite rods to the CBT screws, eschewing the use of rod connectors. This can prevent stress concentration and ensure a more uniform stress distribution across the primary rods. The current study found that the 4R-TT/CBT configuration exhibited the lowest stress on the rods (144.51–219.75 MPa) and demonstrated a substantial reduction (0.2%–21.0%) in all the loading directions compared to the 2R-TT (144.87–278.02 MPa) and 4R-TT (155.78–277.78 MPa) configurations. Since a lower stress on the rods is associated with a reduced risk of RF, it can be hypothesized that the 4R-TT/CBT configuration may be capable of reducing the rate of RF in comparison to the 2R-TT and 4R-TT configurations.

Theoretically, RF should occur at the location of maximum von Mises stress. In PSO cases, the osteotomy site itself induces instability of the spine and leads to a substantial increase in rod strain at the PSO level (Seyed Vosoughi et al., 2019). Previous clinical studies suggested that most of RF occurred at the PSO site and the lumbosacral junction in ASD patients treated by two-rods configurations (Hyun et al., 2014; Dickson et al., 2014). This is consistent with the results of the current study, which suggested that the maximum von Mises stress on the rods in the 2R-TT configuration were found to be adjacent to the PSO level in flexion and left axial rotation and were transferred to the location adjacent to the L5 pedicle screws in extension and left bending. The maximum von Mises stress on the rods in the 4R-TT configuration was adjacent to the connectors where the satellite rods end. This can be attributed to transition in the rigidity from two-rods segment to one-rod segment, which cause the stress concentration adjacent to connectors. These finding give possible explanation to the high rate of RF on primary rods adjacent to connectors in standard satellite-rod configurations (Shen et al., 2018). In the 4R-TT/CBT configuration, the von Mises stress at the at the PSO level was shared by multiple rods. And the maximum von Mises stress on the rods was transferred to the location adjacent to the L5 pedicle screws.

Screw-related failure (pull-out, breakage, and loosening) is a prevalent complication following long-segment fusion for ASD patients, with incidence ranging from 10% to 60%, depending on the definition employed (Marie-Hardy et al., 2020; Yuan et al., 2023). It has been reported that the prevalence of the condition may be elevated in patients with osteoporosis (Marie-Hardy et al., 2020; Yuan et al., 2023; Ponnusamy et al., 2011). Screw-related failure has been demonstrated to result in a range of adverse outcomes, including pain, pseudarthrosis, reoperations, and poor clinical outcomes (Tokuhashi et al., 2008; Bokov et al., 2019). The present study demonstrated that the 4R-TT group exhibited the highest stress on the screws in all directions, despite the reduced stresses on the primary rods. This may be due to the fact that the application of the Multi-RCs increased the overall stiffness of the fixation configuration, and the 4R-TT configuration (similar to the 2R-TT configuration) was linked to each vertebra by only two screws, which led to a corresponding increase in the stress on each screw. However, although the overall stiffness also increased in the 4R-TT/CBT configuration, the stress on each screw was correspondingly reduced because the configuration was linked to each vertebra through four screws. Consequently, the maximum screw stress observed in the 4R-TT/CBT group was minimal in this study. The results of the study suggest that the implementation of 4R-TT/CBT is expected to reduce screw pull-out and breakage, and enhance clinical outcomes.

It has been demonstrated that the maximum screw stress following long segmental fusion for ASD is mainly concentrated at the cephalic-caudal extremities. Consequently, the rates of screw-related complications are highest in the lowest instrumented vertebra (LIV) and the uppermost instrumented vertebra (UIV) (Yuan et al., 2023; Uehara et al., 2017). The results of the present study were consistent with these findings, with the maximum screw stress occurring at the distal end of the screws in all three groups. This finding indicates that techniques such as cemented screws, S2AI screws, or iliac screws should be employed to enhance the distal fixation strength and reduce screw pull-out and breakage.

The results of this study should be considered in the context of its limitations. Initially, the models were simplified. The spinal ligaments were modeled as linear tension-only spring elements, which does not capture their nonlinear force-displacement behavior and pre-strain condition. These simplifications may affect the segmental motion and stress distribution, particularly in combined loading scenarios. Furthermore, the mechanical effects of muscle tissue were omitted, which may result in an underestimation of dynamic stability. Secondly, the loading conditions were static and simplified. It should be noted that the study applied static loads only; dynamic or combined loads (for example, walking, bending) were not considered. Furthermore, the effects of fatigue loading on rods and screws were not taken into consideration. Thirdly, the contacts between rods, screws and connectors were modelled as ‘bonded’ (no relative motion), which can exaggerate stress concentrations. Furthermore, the frictional or viscoelastic behavior exhibited at the interface was not considered in the overall analysis. Fourthly, immediate post-surgical fusion was assumed, with stiffness changes during bone healing being ignored. This cannot reflect the early postoperative phase when the osteotomy surfaces are not yet fused. And effects of interbody cages or bone grafts on stability were not evaluated. Fifthly, long segment fusion of ASD is frequently employed in conjunction with S2AI screws and iliac screws in clinical treatment; however, in order to control for variables, models using S2AI or iliac screws were not constructed in this study, and the effects of the pelvic screws on fixation were not explored. Sixthly, although the 4R-TT/CBT configuration exhibited the most rigid fixation strength and reduced the stress on both rods and screws, the double-trajectory technique is technically challenging and multi-RCs have potential interference with bone fusion.

## Conclusion

The FE study evaluated the biomechanical performance of a novel fixation configuration combining double-trajectory technique with Multi-RCs (4R-TT/CBT) in lumbar PSO models under normal and osteoporotic conditions. The 4R-TT/CBT configuration demonstrated superior mechanical stability, exhibiting the smallest ROM in all loading directions compared to 2R-TT and 4R-TT configurations in both normal and osteoporotic models. It also demonstrated a substantial decrease in von Mises stress in rods and screws, suggesting a reduced probability of RF and screw-related failures. The findings highlighted the clinical potential of 4R-TT/CBT for reducing mechanical complications, particularly in osteoporotic patients requiring PSO and long segment fusion. The present study provides the first biomechanical evidence supporting the combined use of double-trajectory technique and Multi-RCs in patients requiring lumbar PSO and long-segment fusion. Future studies should integrate multi-scale modelling, dynamic experimental validation, and the impact of cages and pelvic screws to bridge biomechanical predictions and surgical optimization.

## Data Availability

The original contributions presented in the study are included in the article/Supplementary Material, further inquiries can be directed to the corresponding authors.
